# Lymph node shape in computed tomography imaging as a predictor for axillary lymph node metastasis in patients with breast cancer

**DOI:** 10.3892/etm.2014.1787

**Published:** 2014-06-16

**Authors:** GORO KUTOMI, TOUSEI OHMURA, FUKINO SATOMI, TOMOKO TAKAMARU, HIROAKI SHIMA, YASUYO SUZUKI, SEIKO OTOKOZAWA, HITOSHI ZEMBUTSU, MITSURU MORI, KOICHI HIRATA

**Affiliations:** 1 First Department of Surgery, School of Medicine, Sapporo Medical University, Sapporo, Hokkaido 060-8543, Japan; 2 Department of Public Health, School of Medicine, Sapporo Medical University, Sapporo, Hokkaido 060-8556, Japan

**Keywords:** breast cancer, computed tomography, lymph node shape

## Abstract

The aim of the present study was to evaluate whether preoperative computed tomography (CT) is a useful modality for the diagnosis of axillary lymph node metastasis. The axillary lymph node status was examined in patients with primary breast cancer who had undergone surgery. In total, 75 patients were analyzed with preoperative contrast CT images, following which the patients underwent an intraoperative sentinel lymph node biopsy to determine possible predictors of axillary lymph node metastasis. The lymph node shape was classified into three groups, which included fat-, clear-and obscure-types. Multivariate analysis revealed that clear-type lymph nodes in preoperative contrast CT imaging may be an independent predictor of lymph node metastasis (odds ratio, 15; P=0.003). Therefore, the results indicated that preoperative CT examination is useful to predict axillary lymph node metastasis.

## Introduction

Axillary lymph node excision in breast cancer was previously the standard optimal surgical procedure for breast cancer. However, currently this procedure is not always essential since the status of axillary lymph node metastasis can be predicted by an intraoperative sentinel lymph node biopsy (SNB) (1). Despite this development, a number of institutions in Japan perform lymph node excision for cases demonstrated to be negative by intraoperative SNB. Thus, axillary lymph node dissection tends to be unnecessary, particularly in a number of patients with early stage breast cancer (2).

Axillary lymph node metastasis is a multifactorial event, and several clinicopathological factors have been reported as predictors of lymph node metastasis in breast cancer (3). However, since only a few methods exist for precisely predicting the axillary lymph node metastasis of an individual patient with breast cancer, a number of patients may not receive appropriate treatment for such metastasis.

The development of diagnostic imaging systems has facilitated the evaluation of axillary lymph node metastasis prior to surgery for breast cancer (4). Computed tomography (CT) is one of the representative modalities that can be used to evaluate the lymph node status, and is commonly used in hospitals due to its noninvasive and inexpensive characteristics. However, the number of studies investigating the clinical usefulness of CT in determining the axillary lymph node status is limited (5).

Therefore, the aim of the present retrospective study was to examine whether contrast CT imaging for the preoperative evaluation of the axillary lymph node status was a clinically useful modality.

## Materials and methods

### Patients

A total of 75 patients with primary breast cancer that had undergone surgical treatment at the First Department of Surgery of Sapporo Medical University (Sapporo, Japan) between 2009 and 2010 were recruited for the study. The clinical data from the Medical Records Department were retrospectively obtained. Written informed consent was required from all patients. All the patients were Japanese females that had been pathologically diagnosed with invasive ductal carcinoma without distant dissemination by whole body CT and bone scintigraphy. In this department, preoperative contrast CT is normally performed.

Data on clinical information were confirmed from the medical records of the patients and are shown in Table I. Tumor status was classified according to UCLA-integrated staging system classification with tumor, node and metastasis categories (6). The expression of the estrogen receptor or progesterone receptor was designated as positive when positive staining was observed and a total Allred score of ≥3 was achieved. Tumors that were immunohistochemically scored 2+ or 3+ and were fluorescence *in situ* hybridization-positive, were regarded as HER2-positive (7). Patients were classified into the following two groups: Group A consisted of patients who had been diagnosed as negative by SNB, while group B comprised patients who had been diagnosed as axillary lymph node metastasis-positive.

### Evaluation of axillary lymph nodes by preoperative contrast CT

**Figure 1:**
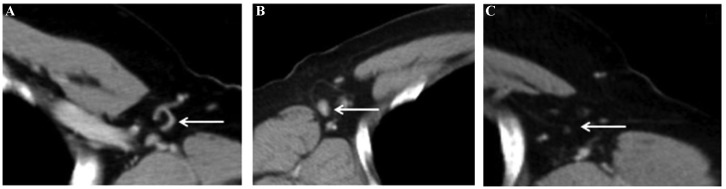
CT images showing (A) fat-, (B) clear-and (C) obscure-type axillary lymph nodes. CT, computed tomography.

Although the axillary lymph nodes were not palpable in any patient, enhanced whole body CT (Aquilion 64; Toshiba, Tokyo, Japan) with contrast was preoperatively performed since this is the standard procedure in Japan. A helical CT unit (64-slice CT system; Light Speed VCT vision; GE Healthcare, Milwaukee, WI, USA) was used for the evaluation of the axillary lymph nodes. The patients were in a supine position and raised their arms during the CT examination. CT images of the axillary lymph nodes were obtained as 2-mm slices through the axilla. The most caudally located enhanced lymph nodes were considered to be the sentinel lymph nodes. Lymph node size and shape were evaluated, as well as the Hounsfield units (HU) of the axillary lymph nodes in the CT images. The average of the region of interest (ROI) was used to evaluate the HU as a CT score. Lymph node shapes were classified into three groups, according to a previous study (8). Nodes with an internal fat concentration were classified as the fat-type (Fig. 1A), those with a size of ≥10 mm that appeared as rounded nodes without any internal fat were classified as the clear-type (Fig. 1B), while the nodes with unclear borders were classified as the obscure-type (Fig. 1C).

### SNB

Prior to the initiation of surgery, 3–5 ml indigo carmine was injected into the peritumor, as well as subcutaneous and intradermal portions of the areola. Sentinel lymph nodes were located following massaging the expected area for 2–3 min. All the sentinel lymph nodes identified were sliced into 2-mm sections and stained with hematoxylin and eosin. A surgeon conducted the SNB, while a pathologist evaluated the specimens during the surgery. Finally, SNB specimens were embedded in paraffin and evaluated.

### Statistical analysis

Analysis of the continuous variables, including age, tumor size, lymph node size and the CT score, was conducted with the t-test, whereas the χ^2^ test was applied for the categorical variables (Table I). For the logistic regression analysis, odds ratios and 95% confidence intervals (CIs) were calculated following adjustment for age. All the statistical analyses and corresponding P-values were two-sided, and P<0.05 was considered to indicate a statistically significant difference. All statistical calculations were performed using JMP version 9.0 software (SAS Institute, Cary, NC, USA).

## Results

### Characteristics of the patients

A total of 75 patients who had received adequate treatment for primary breast cancer were analyzed in the study (Table I). A mastectomy was performed for 61% of the population.

Patients were classified into the following two groups according to the histological diagnosis from the SNB. Group A (n=56) patients were diagnosed as axillary lymph node metastasis-negative by SNB, while group B (n=19) patients were diagnosed as axillary lymph node metastasis-positive.

### Difference in the distributions of the possible predictors of axillary lymph node metastasis

Differences in the menopausal status, histological type, tumor size, axillary lymph node size, axillary lymph node shape in contrast CT and CT scores (the average of the ROI) were analyzed between groups A and B (Table II). The menopausal status, tumor size, axillary lymph node size, axillary lymph node shape and CT score exhibited statistically significant differences when comparing the two groups (Table II). In addition, the ratio of the premenopausal group was higher in group B compared with group A (P=0.034), and the primary tumor size, axillary lymph node size and CT score (ROI) were larger in group B compared with group A (P=0.034, P=0.0007 and P<0.0001, respectively). Furthermore, of the 56 patients in group A, fat-, clear- and obscure-type lymph nodes were observed in 17 (30.4%), 8 (14.3%) and 31 cases (55.3%), respectively. By contrast, fat-, clear- and obscure-type lymph nodes were identified in two (10.5%), 14 (73.7%) and three cases (15.8%) in group B, respectively, indicating that there were statistically significant differences (P<0.0001) in the distribution of the lymph node shapes in preoperative contrast CT between the two groups (Table II).

### Identification of the predictors for axillary lymph node metastasis

To identify the risk factors for axillary lymph node metastasis, logistic regression analysis of the menopausal status, tumor size, axillary lymph node size, axillary lymph node shape and CT score was conducted since the aforementioned predictors significantly differed between the groups (Table III). In univariate analysis, the menopausal status, axillary lymph node size, obscure-type lymph nodes, clear-type lymph nodes and the CT score were demonstrated to be predictors of lymph node metastasis (P=0.036, P=0.01, P=0.006, P<0.001 and P=0.013, respectively, with 95% CIs of 0.11–0.93, 0.0062–0.64, 0.04–0.58, 4.7–60 and 0.15–6.0, respectively). In addition, with regard to the multivariate analysis, clear-type axillary lymph nodes were shown to be significantly associated with axillary lymph node metastasis following adjustment for the menopausal status, axillary lymph node size, obscure-type lymph nodes and the CT score (P=0.003; 95% CI, 2.5–89; Table III), indicating that the axillary lymph node shape in preoperative contrast CT imaging was an independent indicator of axillary lymph node metastasis (SNB-positive).

## Discussion

Lymph node metastasis is an important factor that affects the prognosis and management of patients with breast cancer (9). Although the axillary lymph nodes should be dissected for patients who are considered to be axillary lymph node-positive, lymph node dissection often causes complications, including arm edema, motor disturbance of the arm and axillary numbness (10–12). Therefore, axillary lymph node dissection should be performed only following consideration of whether the procedure is essential in each patient with breast cancer. In the present study, to identify preoperative predictors for axillary lymph node metastasis, the association of possible predictors and preoperative contrast CT observations were investigated with axillary lymph node metastasis. Axillary lymph node shape in preoperative contrast CT imaging was found to be an independent predictor of metastasis. As shown in Table III, multivariate analysis indicated that clear-type axillary lymph nodes in contrast CT were likely to be a predictor of metastasis (odds ratio, 15; P=0.003; 95% CI, 2.5–89). Although soybean-shaped lymph nodes have been reported to be significantly metastatic and ‘C’-shaped and ring-like lymph nodes are more likely to be nonmetastatic in contrast-enhanced CT imaging (8), the clear- and fat-type lymph nodes defined in the present study were demonstrated to correspond to the former and latter, respectively. The pathological association between the lymph node shape in contrast CT and the localization of cancer cells in lymph nodes has not yet been established. Thus, further clinicopathological investigations may clarify how the localization of cancer cells in lymph nodes influences their imaging or shape in contrast CT.

Tumor size has been reported to be one of the main predictors of axillary lymph node metastasis in several studies (13–16). Although statistically significant differences were observed in the distribution of tumor size between groups A and B (Table II), tumor size was not found to be an independent predictor for axillary lymph node metastasis in the present study (Table III). However, future studies with larger sample sizes are required to validate the association between tumor size and lymph node metastasis, since 50% of the tumors in the present study were small (<20 mm). SNB has become a standard procedure, and preoperative evaluation of the axillary lymph nodes based on imaging modalities is considered to be important for selecting appropriate breast cancer treatment (16,17). Several diagnostic imaging modalities have been used for the preoperative diagnosis of the sentinel lymph node status. Ultrasonography, magnetic resonance imaging and multidetector CT have been reported to be useful imaging systems to preoperatively evaluate the lymph node status (18–20).

Lymph node size was also shown to be associated with lymph node metastasis through univariate analysis; however, lymph node size is unlikely to be an independent predictor according to the results from the multivariate analysis (Table III). In the present study, univariate analysis demonstrated that the CT score (ROI) was a predictor of lymph node metastasis, indicating that high contrast lymph nodes on CT images, which may be a consequence of vessel development in the lymph nodes, may be associated with metastasis (Table III). These observations indicate that the evaluation of the lymph node status by preoperative contrast CT may support the intraoperative diagnosis by SNB.

In Japan, CT examinations are indispensable for the preoperative metastatic search, and are conducted in all institutions. CT is also considered to be very important for preoperative sentinel lymph node examination. The results of the present study indicate that preoperative CT examinations are useful in predicting axillary lymph node metastasis, and can provide supportive information for intraoperative sentinel lymph node diagnosis. Although further large-scale studies are required to validate these results, the observations of the present study provide useful information for identifying predictors of axillary lymph node metastasis, and may aid surgeons to determine appropriate surgical strategies for individual patients with breast cancer.

## Figures and Tables

**Table I:** Clinical characteristics of the 75 patients with breast cancer.

Characteristics	Patients
Mean age, years (range)	
Total (n=75)	56 (35–84)
Pre-menopause (n=28)	54 (32–60)
Post-menopause (n=47)	60 (40–82)
pT^a^, n (%)	
pTis	14 (18.7)
pT1	23 (30.6)
pT2	38 (50.7)
HR status, n (%)	
ER(+), PgR(+)	40 (53.4)
ER(+), PgR(−)	19 (25.3)
ER(−), PgR(+)	7 (9.3)
ER(−), PgR(−)	9 (12.0)
HER2 status, n (%)	
Positive	11 (14.7)
Negative	64 (85.3)
pN^a^, n (%)	
pN0	56 (74.7)
pN1	19 (25.3)
pN2	0 (0)
Surgery, n (%)	
Breast-conserving	28 (37.3)
Mastectomy	47 (62.7)

a UCLA-integrated staging system classification with tumor, node and metastasis categories (2002).

HR, hormone receptor; ER, estrogen receptor; PgR, progesterone receptor.

**Table II:** Differences in the distributions of possible predictors for positive SNB.

Characteristics	Group A (n=56)	Group B (n=19)	P-value
Menopause (pre/post), n	17/39	11/08	0.034
Tumor size^b^, cm	1.55±0.15	2.19±0.26	0.034
Axillary lymph node size^b^, cm	0.56±0.05	0.92±0.09	0.0007
Axillary lymph node shape in contrast CT (fat/clear/obscure), n	17/08/31	2/14/3	<0.0001
CT score (ROI)^a^,^b^	0.16±21.6	31.4±31.9	<0.0001

a Average of the ROI.

b Results are expressed as the mean ± standard deviation.

SNB, sentinel lymph node biopsy; CT, computed tomography; ROI, region of interest.

**Table III:** Univariate and multivariate analyses of the predictors of SNB.

	Univariate analysis			Multivariate analysis		
		
Predictors	Odds ratio	95% CI	P-value	Odds ratio	95% CI	P-value
Tumor size (≥2 cm, <2 cm)	0.84	0.29–2.39	0.74	0.45	0.10–1.8	0.26
Lymph node size (≥0.5, <0.5)	0.12	0.0062–0.64	0.01	0.16	0.0071–1.6	0.12
Shape						
Obscure	0.15	0.040–0.58	0.006	0.30	0.056–1.6	0.15
Clear	17	4.7–60	<0.001	15	2.5–89	0.003
Fat	0.27	0.56–1.3	0.102	0.16	0.025–1.1	0.06
CT score (ROI^a^; ≥0, <0)	0.22	0.047–0.74	0.013	0.95	0.15–6.0	0.95

a Average of the ROI.

Values in brackets are the optimal cut-off point defined using a receiver operating characteristic curve. CI, confidence interval; SNB, sentinel lymph node biopsy; CT, computed tomography; ROI, region of interest.
